# One day in Denmark: whole-genome sequence-based analysis of *Escherichia coli* isolates from clinical settings

**DOI:** 10.1093/jac/dkaf028

**Published:** 2025-01-30

**Authors:** Ana Rita Rebelo, Valeria Bortolaia, Pimlapas Leekitcharoenphon, Dennis Schrøder Hansen, Hans Linde Nielsen, Svend Ellermann-Eriksen, Michael Kemp, Bent Løwe Røder, Niels Frimodt-Møller, Turid Snekloth Søndergaard, John Eugenio Coia, Claus Østergaard, Henrik Westh, Frank M Aarestrup

**Affiliations:** Technical University of Denmark, National Food Institute, Kongens Lyngby, Denmark; Technical University of Denmark, National Food Institute, Kongens Lyngby, Denmark; Technical University of Denmark, National Food Institute, Kongens Lyngby, Denmark; Department of Clinical Microbiology, Herlev and Gentofte Hospital, Herlev, Denmark; Department of Clinical Microbiology, Aalborg University Hospital, Aalborg, Denmark; Department of Clinical Medicine, Aalborg University, Aalborg, Denmark; Department of Clinical Microbiology, Aarhus University Hospital, Aarhus, Denmark; Department of Clinical Microbiology, Odense University Hospital, Odense, Denmark; Department of Clinical Microbiology, Slagelse Hospital, Slagelse, Denmark; Department of Clinical Microbiology, Rigshospitalet, København, Denmark; Department of Clinical Microbiology, Hospital of Southern Jutland, Sønderborg, Denmark; Department of Clinical Microbiology, Hospital of South West Jutland, Esbjerg, Denmark; Department of Clinical Microbiology, Vejle Hospital, Vejle, Denmark; Department of Clinical Microbiology, Hvidovre Hospital, Copenhagen University Hospital—Amager and Hvidovre, Hvidovre, Denmark; Department of Clinical Medicine, University of Copenhagen, Copenhagen, Denmark; Technical University of Denmark, National Food Institute, Kongens Lyngby, Denmark

## Abstract

**Background:**

WGS can potentially be routinely used in clinical microbiology settings, especially with the increase in sequencing accuracy and decrease in cost. *Escherichia coli* is the most common bacterial species analysed in those settings, thus fast and accurate diagnostics can lead to reductions in morbidity, mortality and healthcare costs.

**Objectives:**

To evaluate WGS for diagnostics and surveillance in a collection of clinical *E. coli*; to examine the pool of antimicrobial resistance (AMR) determinants circulating in Denmark and the most frequent STs; and to evaluate core-genome MLST (cgMLST) and SNP-based clustering approaches for detecting genetically related isolates.

**Methods:**

We analysed the genomes of 699 *E. coli* isolates collected throughout all Danish Clinical Microbiology Laboratories. We used rMLST and KmerFinder for species identification, ResFinder for prediction of AMR, and PlasmidFinder for plasmid identification. We used Center for Genomic Epidemiology MLST, cgMLSTFinder and CSI Phylogeny to perform typing and clustering analysis.

**Results:**

Genetic AMR determinants were detected in 56.2% of isolates. We identified 182 MLSTs, most frequently ST-69, ST-73, ST-95 and ST-131. Using a maximum 15-allele difference as the threshold for genetic relatedness, we identified 23 clusters. SNP-based phylogenetic analysis within clusters revealed from 0 to 13 SNPs, except two cases with 111 and 461 SNPs.

**Conclusions:**

WGS data are useful to characterize clinical *E. coli* isolates, including predicting AMR profiles and subtyping in concordance with surveillance data. We have shown that it is possible to adequately cluster isolates through a cgMLST approach, but it remains necessary to define proper interpretative criteria.

## Introduction

*Escherichia coli* is a commensal bacterium present in the gastrointestinal tract of humans and other animals.^1^ However, this species also possesses pathogenic potential, and is frequently associated with diarrhoeal diseases, urinary tract infections and bloodstream infections.^2^ Typing methods are helpful predictors of *E. coli* pathogenicity because specific serotypes and sequence types (STs) are associated with certain clinical manifestations or public health implications.^3^ Famously, the extraintestinal clone belonging to ST-131 is globally disseminated and presents an MDR profile, demanding timely detection through notification and surveillance systems.^4–6^ Other STs have been associated with community outbreaks, such as the entero-aggregative-haemorrhagic ST-678 responsible for a large foodborne disease outbreak in Germany,^7^ and the enterohaemorrhagic ST-11 detected in the UK.^8^

Determination of STs is classically performed by MLST, which consists of amplifying seven target housekeeping genes through PCR and sequencing the obtained fragments. The sequences of each gene are compared with reference databases and their specific alleles are determined, and each allelic profile corresponds to a certain ST.^9^ The most common combination of genes used for *E. coli* MLST is the Achtman scheme targeting the genes *adk*, *fumC*, *gyrB*, *icd*, *mdh*, *purA* and *recA*,^3^ but others, such as the Pasteur scheme, also exist.^10,11^ Nowadays, with the advent of WGS technologies and an increase in sequencing accuracy, MLST is often performed through bioinformatics analysis of the complete bacterial genomes.^12^ One of the most frequently used databases hosting MLST schemes is PubMLST (https://pubmlst.org/).^13^

A more recent approach for typing *E. coli* is the core-genome MLST scheme (cgMLST).^14^ cgMLST typing is performed in the same way as MLST, but the cgMLST scheme contains thousands of conserved loci (https://www.cgmlst.org/ncs/) instead of only seven housekeeping genes, thus presenting a higher discriminatory power.^15^ Furthermore, cgMLST allows for clustering of related isolates by comparing the dissimilarity between the alleles assigned to each locus. cgMLST quality and interpretative thresholds are not currently harmonized. It has been suggested that a threshold of 0.0105 should correspond to the maximum accepted dissimilarity to consider that isolates are genetically related, which translates to 26 alleles if using the EnteroBase *E. coli* cgMLST scheme of 2513 loci.^16^ To interpret the difference in number of alleles between genomes, the majority of the loci in the scheme should be assigned a specific allele, and a study has reported that, on average, 97.1% of alleles were assigned for *E. coli* isolates.^17^

cgMLST approaches, either using the EnteroBase scheme or schemes designed in the context of each study, have been used to analyse and properly detect clustering of Shiga toxin-producing *E. coli* (STEC) recovered from food or food-producing animals.^18,19^ These approaches have been able to clarify an outbreak of carbapenem-resistant Enterobacterales in a German hospital, detecting three clusters and one singleton among 19 *E. coli* isolates, with maximum of eight allele differences between isolates in the same clusters.^20^ A paediatric outbreak of STEC in France was also solved by employing cgMLST analysis with a defined threshold of fewer than 10 different alleles between isolates, in combination with other methods, including serotyping and sequence typing through bioinformatics tools from the Center for Genomic Epidemiology (CGE).^21^

For investigation of closely related isolates, SNP-based cluster analysis has a higher discriminatory power than cgMLST approaches. Reference-based SNP analysis starts with the alignment of the query genomes against a reference genome, and identification of a core genome that corresponds to all common regions amongst the genomes and the reference. All differences in nucleotides (the SNPs) within that core genome are then investigated through variant calling. Thus, if most regions of the query genomes are comparable, the method provides higher resolution regarding genomic variations than gene-by-gene approaches, because it is not restricted to the specific loci included in a scheme.^22,23^ Very closely related isolates will present, simultaneously, high percentages of aligned genomes and a low number of SNP differences, and thresholds from 5 to 20 SNPs have been proposed for inferring clonal and epidemiological relatedness between *E. coli* isolates.^24,25^ However, because results are dependent on the chosen reference genome and on the underlying algorithms and statistical methods of each bioinformatics tool, defining universal thresholds is difficult, even within the same species or subtype.^17,26,27^

As for cgMLST, SNP-based phylogenetic analysis has been applied to review or resolve *E. coli* outbreaks and events of transmission. For example, epidemiologically linked STEC from foodborne and zoonotic outbreaks have been adequately clustered, with maximum distances of three and five SNPs, respectively, and the analyses allowed for source prediction and separation from other unrelated outbreak events.^28,29^ In another example, prospective screening in haematology departments identified 10 *E. coli* clusters across 20 patients using threshold values of 17 SNP differences, thus providing evidence of *E. coli* transmission during routine care.^30^

In our study we sequenced and analysed the genomes of a large collection of *E. coli* isolates recovered in 1 day from all Danish clinical microbiology laboratories, in order to determine the applicability of WGS technologies in clinical settings for diagnostics purposes. We performed species identification, predicted antimicrobial resistance (AMR) profiles, detected plasmid replicons and performed bacterial subtyping. Furthermore, we analysed the adequacy of cgMLST and SNP-based clustering approaches for public health surveillance purposes.

## Materials and methods

Previously, we collected and sequenced all clinically relevant isolates (*n* = 2009) processed by the 11 Danish Clinical Microbiology (DCM) laboratories in 1 day.^31^ As described in that study, WGS was performed using the Illumina NextSeq 500 platform and according to the species identification provided by the DCM laboratories (obtained through MALDI-TOF MS) and the *in silico* taxonomic analysis (using rMLST and KmerFinder), 707 isolates belonged to the genus *Escherichia*.

For the current study, we repeated the bioinformatics taxonomic analysis of the 707 *Escherichia* spp. isolates using the Danish National Supercomputer for Life Sciences (https://www.computerome.dk). Updated versions of KmerFinder (April 2022) and rMLST (April 2022) were used, and led to classification of 699 isolates as *E. coli.* The remaining eight isolates belonged to other species or revealed contamination and thus were not included in this study (Table S1, available as Supplementary data at *JAC* Online).

For the 699 *E. coli* isolates, we detected AMR determinants with ResFinder 4.0 (https://cge.food.dtu.dk/services/ResFinder/) using the threshold values of minimum accepted identity of 90% and minimum accepted coverage of 60%, which corresponded to the default thresholds proposed in the online webtool.^32^ We investigated acquired AMR genes (ARGs) and chromosomal point mutations (PMs) mediating resistance to the following antimicrobial agents or classes: β-lactam agents, chloramphenicol, ciprofloxacin, colistin, gentamicin, sulfamethoxazole, tetracycline and trimethoprim. We identified plasmid replicons using PlasmidFinder 2.1 (https://cge.food.dtu.dk/services/PlasmidFinder/) with the threshold values of minimum accepted identity of 90% and minimum accepted coverage of 60%, which corresponded to the default thresholds proposed in the online webtool.^33^ STs were determined using CGE MLST (https://cge.food.dtu.dk/services/MLST/) with the scheme *E. coli* #1 (Achtman).^10^ All three programs were executed in a batch using the assembled genomes in *fasta* format.

cgMLST STs were determined using cgMLSTFinder (https://cge.food.dtu.dk/services/cgMLSTFinder/) with cgMLST loci sequences retrieved from EnteroBase (https://enterobase.warwick.ac.uk).^14,34,35^ Trimmed raw reads in *fastq* format were used as input data. The pairwise dissimilarities (distances) between genomes were identified based on the allele profile using ‘Gower’ distance method from the ‘cluster’ package in R. The cgMLST tree was constructed from the distance matrix using hierarchical clustering from the ‘ape’ package in R. The distances were converted into the number of different alleles by multiplying the obtained percentages by the number of loci present in the cgMLST scheme (*n* = 2513). We applied an exploratory threshold of 15 alleles to infer genetic relatedness between isolates. We used the Interactive Tree of Life (iTOL) (https://itol.embl.de/) for visualization of results. We used CSI Phylogeny (https://cge.food.dtu.dk/services/CSIPhylogeny/) to determine SNPs between isolates belonging to the clusters identified through the cgMLST analysis, using all default threshold values found in the online tool, and using trimmed raw reads in *fastq* format.^36^ Each cluster was analysed independently using one of the genomes of that cluster as reference, specifically the one with the lowest number of contigs and thus higher quality.

A subset of 16 isolates were later re-analysed through submission to EnteroBase in December 2024 due to unresolved results obtained with either cgMLSTFinder and/or CGE MLST.

Raw sequence data have been submitted to the European Nucleotide Archive (http://www.ebi.ac.uk/ena) under study accession no. PRJEB37711. A complete list of genomic sequence data is available in Table S1, as well as metadata and the complete output from the bioinformatics tools.

## Results

### Bacterial isolates and AMR determinants

Each DCM laboratory provided between 1.3% (*n* = 9) and 27.8% (*n* = 194) of the 699 *E. coli* isolates (Table 1). The most prevalent sample source of *E. coli* isolates was urine (*n* = 639; 91.4%), and all other sources presented much lower prevalence, from 0.1% to 3.1% (Table 1).

**Table 1.** Distribution of the 699 *E. coli* isolates according to sample source and DCM laboratory of origin

		DCM laboratory											Totals	
		F4	F2	F1	F5	F3	F6	F10	F9	F8	F11	F7	Total	(%)
Sample source	Urine	190	120	86	64	53	51	29	12	16	12	6	639	91.4
	Blood	2	5	4	1	1	1	4	2		2		22	3.1
	Stool or rectum	1	3	2	10		1						17	2.4
	Respiratory system				3	1	1	1	2		1		9	1.3
	Skin or soft tissue				2							1	3	0.4
	Abdomen	1											1	0.1
	Bone or joint											1	1	0.1
	Undetermined		2	1	1		1		1			1	7	1.0
Totals	Total	194	130	93	81	55	55	34	17	16	15	9	699	100
	(%)	27.8	18.6	13.3	11.6	7.9	7.9	4.9	2.4	2.3	2.1	1.3	100	

**Figure 1.**
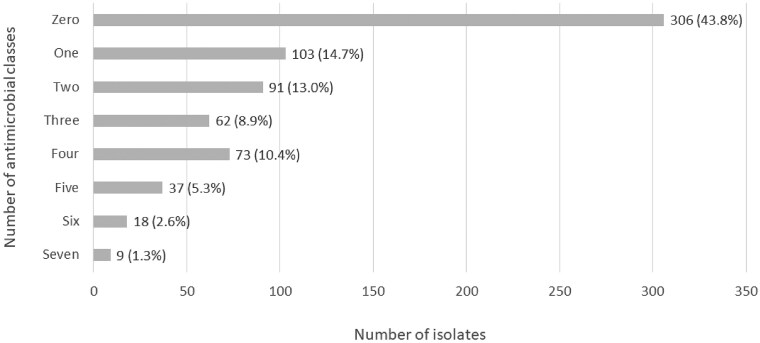
Number (and percentage) of *E. coli* isolates harbouring ARGs or PMs mediating resistance to antimicrobial agents belonging to one, two, three, four, five, six or seven antimicrobial classes.

ARGs or chromosomal PMs were detected in 393 isolates (56.2%) (Table S1). Of the isolates harbouring genetic determinants of resistance, the highest proportion (*n* = 103; 14.7%) presented ARGs or PMs mediating resistance to agent(s) within one single antimicrobial class. However, we also detected cases of isolates harbouring ARGs or PMs mediating resistance to two, three, four, five, six and all seven analysed classes simultaneously (Figure 1). The antimicrobial agents for which most genetic resistance determinants were detected were the β-lactam agents (*n* = 280; 40.1%), followed by sulfamethoxazole (*n* = 223; 31.9%), trimethoprim (*n* = 198; 28.3%), tetracycline (*n* = 185; 26.5%) and ciprofloxacin (*n* = 153; 21.9%). ARGs associated with carbapenem resistance were not detected in any of the isolates. From all the identified ARGs, none presented a percentage of identity below 96% (Table S2). Simultaneously, the majority of the ARGs had a percentage of coverage higher than 90%, with some exceptions, including seven of the *sul* genes identified having coverage between 61% and 76%, 10 of the *catB3* genes having a coverage of 70%, and for seven isolates different variants of *bla*_TEM_ were assigned to the same locus with percentages of coverage from 61% to 90%. Table 2 provides details regarding the specific ARGs or PMs detected in the *E. coli* isolates.

**Table 2.** Genetic determinants of AMR detected in the 699 *E. coli* isolates, according to target antimicrobial agents

Antimicrobials	Number (%) of isolates	Description
β-Lactam agents^a^	280 (40.1)	*bla*_TEM_ (*n* = 235), *bla*_CTX-M_ (*n* = 14), *bla*_SHV_ (*n* = 7), *bla*_OXA_ (*n* = 4), *bla*_DHA_ (*n* = 1), *bla*_CARB_ (*n* = 1), *bla*_CTX-M_ and *bla*_OXA_ (*n* = 9), *bla*_TEM_ and *bla*_CTX-M_ (*n* = 6), *bla*_TEM_ and *bla*_OXA_ (*n* = 1), *bla*_TEM_ and *bla*_DHA_ (*n* = 1), *ampC* 32T > A (*n* = 1)
Chloramphenicol	49 (7)	*catA1* (*n* = 22), *catB3* (*n* = 13), *cmlA1* (*n* = 8), *floR* (*n* = 6)
Ciprofloxacin	153 (21.9)	Single PM in *gyrA*/*parE*/*parC* (*n* = 65), 2 PMs in *gyrA*/*parE*/*parC* (*n* = 9), more than 2 PMs in *gyrA*/*parE*/*parC* (*n* = 51), *qnrS1* (*n* = 9), *qnrS1* and PMs (*n* = 2), *qnrB4* (*n* = 2), *qnrB19* (*n* = 2), *qnrD1* (*n* = 1), *qnrS2* (*n* = 1), *aac(6′)-Ib-cr* (*n* = 1), *aac(6′)-Ib-cr* and PMs (*n* = 10)
Colistin	2 (0.3)	*pmrB* V161G (*n* = 2)
Gentamicin	29 (4.1)	*aac(3)-IId* (*n* = 18), *aac(3)-IIa* (*n* = 6), *aac(3)-IV* (*n* = 2), *aac(3)-IVa* (*n* = 1), *ant(2″)-Ia* (*n* = 1), *aac(3)-IId*/*aac(3)-IIa* (*n* = 1)
Sulfamethoxazole	223 (31.9)	*sul1* (*n* = 57), *sul2* (*n* = 98), *sul3* (*n* = 5), *sul1*/*sul2* (*n* = 56), *sul1*/*sul3* (*n* = 2), *sul2*/*sul3* (*n* = 2), *sul1*/*sul2*/*sul3* (*n* = 1), *folP* P64S (*n* = 2)
Tetracycline	185 (26.5)	*tet*(A) (*n* = 99), *tet*(B) (*n* = 84), *tet*(M) (*n* = 1), *tet*(A)/*tet*(B) (*n* = 1)
Trimethoprim	198 (28.3)	*dfrA17* (*n* = 64), *dfrA1* (*n* = 36), *dfrA14* (*n* = 33), *dfrA5* (*n* = 22), *dfrA7* (*n* = 17), *dfrA12* (*n* = 11), *dfrA8* (*n* = 6), *dfrA36* (*n* = 2), *dfrA21* (*n* = 1), *dfrA1*/*dfrA5* (*n* = 3), *dfrA14*/*dfrA17* (*n* = 2), *dfrA1*/*dfrA12* (*n* = 1)

^a^Genes were grouped into families due to the very high diversity of variants and combinations. None of the identified β-lactamases were associated with carbapenem resistance.

### In silico typing and cluster analysis

Through CGE MLST, STs were assigned to 692 isolates (99%) (Table S1). In these, 182 STs were identified, with the most frequent being ST-69 (*n* = 73; 10.4%), followed by ST-73 (*n* = 65; 9.3%), ST-95 (*n* = 53; 7.6%) and ST-131 (*n* = 47; 6.7%). From the remaining seven isolates, five presented an unknown ST. These five isolates were re-analysed in EnteroBase: one isolate did not pass the quality control (and therefore was not assembled or analysed); another isolate received an ST, therefore resolving the result previously obtained with CGE MLST; the three other isolates that originally presented an unknown ST received STs with negative numbers indicating missing or yet unassigned loci, which was in accordance with the results previously obtained with CGE MLST (Table S6). The final two isolates out of the full collection of 699 isolates had been assigned STs with low confidence, and were later re-analysed in EnteroBase: one isolate did not pass the platform’s quality control (and therefore was not assembled or analysed), and the other isolate received an ST therefore resolving the result previously obtained with CGE MLST.

Using cgMLSTFinder, alleles were assigned to more than 90% of all cgMLST loci in 686 isolates (98.1%) (Table S3). For 10 (1.4%) of the remaining 13 isolates, alleles were assigned from 80% to 90% of loci. For the remaining three isolates, alleles were assigned from 74.8% to 75.3% of loci. On average, 98.1% of alleles were assigned. The 13 isolates with fewer than 90% of alleles assigned through cgMLSTFinder were re-analysed in EnteroBase: three isolates did not pass the quality control (and therefore were not assembled nor analysed), and the other 10 isolates received an ST, therefore resolving the results previously obtained with cgMLSTFinder (Table S6).

**Figure 2.**
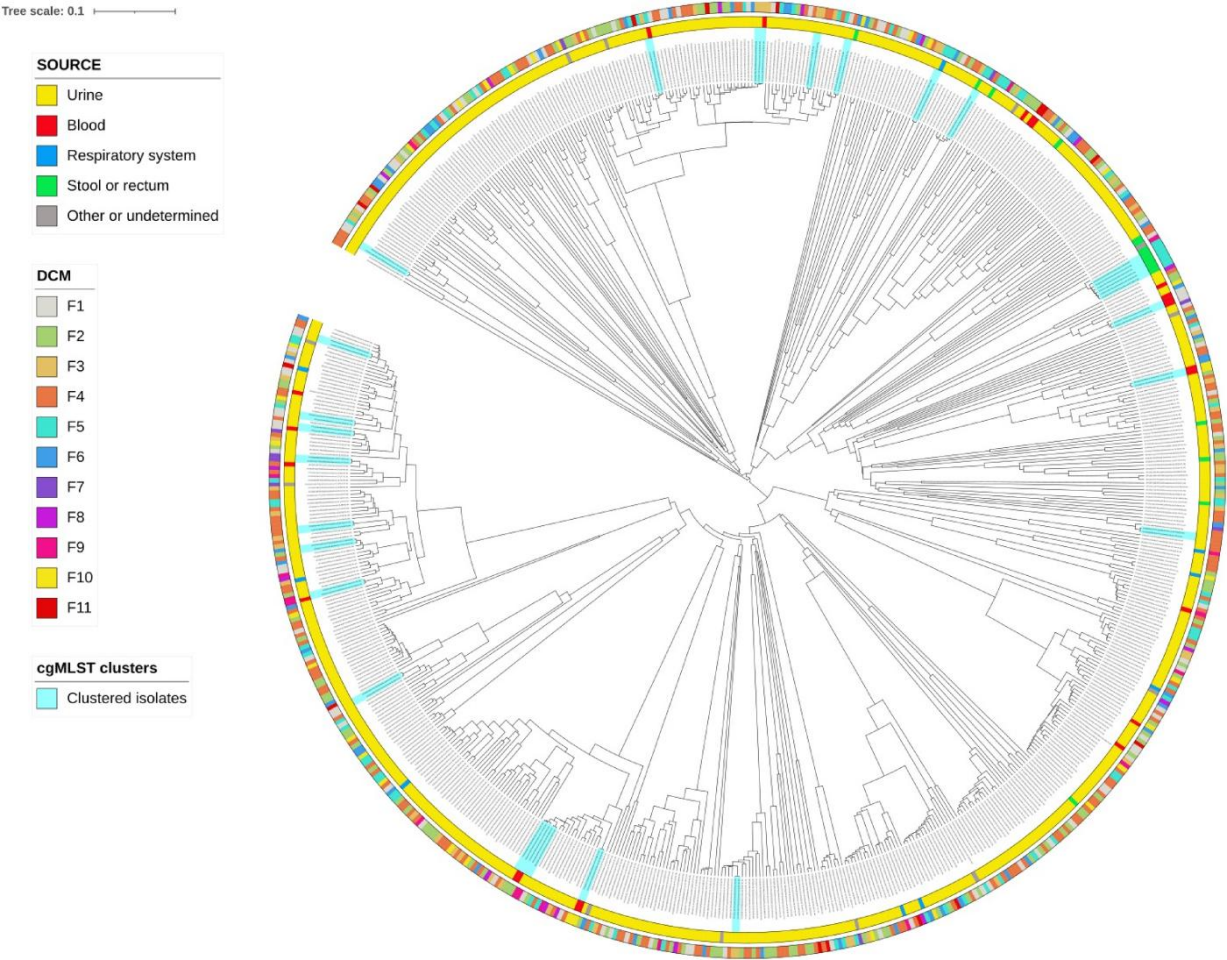
cgMLST tree of the 699 *E. coli* isolates and respective sample source (inner coloured ring) and DCM laboratory of origin (outer coloured ring). The 23 clusters with ≤15 alleles of dissimilarities are highlighted in blue. The high resolution file is available as Figure S1.

The average core-genome dissimilarity from cgMLSTFinder results was 2197 alleles. We observed 23 clusters, containing a total of 36 isolates presenting a dissimilarity equal or inferior to our exploratory threshold of 15 alleles (Table S4). From the 23 clusters, 20 were composed of two isolates, and the remaining three clusters contained more than two isolates (two clusters with three isolates and one cluster with six isolates) (Table 3, Figure 2). The cluster with six isolates (cluster 3) included certain pairs of isolates that exceeded the threshold of 15 alleles dissimilarity, because these isolates presented dissimilarity below the threshold when paired with other isolates belonging to the same cluster. All 36 isolates belonging to the clusters had more than 90% of alleles assigned to the cgMLST loci. All isolates belonging to the same cluster presented the same MLST type. Furthermore, almost all isolates from the same clusters harboured the same ARGs or PMs, except one instance of *tet*(A) detected only in one isolate from a clustered pair (cluster 22). Plasmid distribution in isolates within clusters was slightly more diverse: in 17 clusters the same plasmid replicons were detected in all isolates. However, in four clusters certain replicons were only identified in some of the isolates, specifically the replicon Col(MG828) in clusters 4 and 20, the replicon IncFII in cluster 12, and five out of the six replicons detected in cluster 22.

**Table 3.** Description of the 23 clusters identified through cgMLST with ≤15 alleles of dissimilarities between core genomes

Clusters	Pair of isolates	cgMLST allele difference (*n*)	SNP difference (*n*)	Valid positions in reference (%)	MLST ST	Source	DCM laboratory	ARGs and PMs	Plasmid replicons
Cluster 1	1D-053 1D-700^a^	5	0	95.3	ST-95	**Urine** **Blood**	F2	*sul1*, *dfrA5*	IncFII(29), IncB/O/K/Z, Col156, IncFIB(AP001918)
	1D-699 1D-700	9	0			Blood			
	1D-053 1D-699	10	0			**Urine** **Blood**			
Cluster 2	1D-080 1D-083^a^	3	2	99.7	ST-69	**Blood** **Urine**	F3	*bla*_TEM-1B_, *catA1*, *tet*(B)	IncFIA, IncFII(pSE11), IncI1-I(Alpha), Col156, IncFII, Col(MG828), IncFIB(AP001918)
	1D-083 1D-809	5	0			Urine			
	1D-080 1D-809	6	1			**Blood** **Urine**			
Cluster 3	1D-1200 1D-1199	7	0	98.7	ST-33	Stool/rectum	F5	*bla*_TEM-1B_, *tet*(B)	Col(MP18), Col156, Col(MG828), IncB/O/K/Z, IncFIA, IncFII/IncFII(pCoo)
	1D-1203^a^ 1D-1199	8	0						
	1D-1203 1D-1200	9	1						
	1D-140 1D-1199	10	0						
	1D-1201 1D-1200	11	1						
	1D-1201 1D-1199	12	1						
	1D-1202 1D-1203	12	0						
	1D-140 1D-1203	12	1						
	1D-140 1D-1200	13	0						
	1D-1202 1D-1199	14	0						
	1D-140 1D-1201	16	1						
	1D-140 1D-1202	16	0						
	1D-1201 1D-1203	18	1						
	1D-1200 1D-1202	19	0						
	1D-1201 1D-1202	20	1						
Cluster 4	1D-1384 1D-1385^a^	2	0	99.9	ST-95	Blood	F10	None	**IncFII(29)/IncFIB(AP001918), Col156, Col(MG828) (only 1D-1384)**
Cluster 5	1D-018 1D-502^a^	4	0	99.9	ST-73	Urine	F1	None	Col156
Cluster 6	1D-168^a^ 1D-1342	4	1	99.9	ST-95	Urine	F9	None	IncFII(29), IncFIB(AP001918), Col156
Cluster 7	1D-813 1D-814^a^	4	0	99.9	ST-127	Urine	F3	*bla*_CTX-M-15_	IncFII
Cluster 8	1D-114^a^ 1D-1055	6	0	99.5	ST-91	Urine	F4	None	IncFII/IncFII(pCoo)/IncFIB(AP001918)
Cluster 9	1D-992 1D-1018^a^	6	0	99.9	ST-73	Urine	F4	*sul1*	Col156, IncFII, IncFIB(AP001918)
Cluster 10	1D-156 1D-1277^a^	7	0	99.7	ST-73	Urine	F7	*tet*(B)	IncFII(pRSB107), IncFIA, IncFIB(AP001918)
Cluster 11	1D-831 1D-834^a^	7	0	99.9	ST-69	Urine	F3	*tet*(B), *sul1*, *dfrA17*	IncFII(29), IncFII(pCoo), IncFIB(AP001918), Col156
Cluster 12	1D-019 1D-020^a^	8	111	99.8	ST-73	**Urine** **Blood**	F1	*bla*_TEM-1B_, *tet*(B)	**IncFIA, IncFIB(AP001918), IncFII (only 1D-019)**
Cluster 13	1D-163 1D-1332^a^	8	2	99.8	ST-968	**Urine** **Blood**	F9	None	ColRNAI, Col156
Cluster 14	1D-745 1D-1228^a^	8	12	99.9	ST-2013	Urine	**F2** **F6**	None	None
Cluster 15	1D-1190 1D-1205^a^	8	0	99.8	ST-1434	**Urine** **Stool/rectum**	F5	None	Col440I, Col(pHAD28), IncFII, IncFIB(K), IncX1
Cluster 16	1D-507^a^ 1D-1089	9	13	99.8	ST-69	Urine	**F1** **F4**	None	Col156
Cluster 17	1D-1035^a^ 1D-1053	9	0	99.9	ST-62	Urine	F4	None	IncFII(29), Col156, Col(MG828), IncFIB(AP001918)
Cluster 18	1D-022^a^ 1D-566	10	1	99.7	ST-73	Urine	F1	*bla*_SHV-1_, *sul1*	Col156, IncFII/IncFIB(AP001918)
Cluster 19	1D-171^a^ 1D-599	11	0	96.1	ST-141	Urine	F1	None	IncY
Cluster 20	1D-1124 1D-1160^a^	11	1	99.4	ST-5784	**Respiratory** **Urine**	**F4** **F5**	None	**IncY, IncFIB(K), Col(MG828) (only 1D-1124)**
Cluster 21	1D-009^a^ 1D-014	12	0	99.8	ST-155	Blood	F10	*bla*_TEM-1B_, *tet*(A)	IncFIA, IncFIC(FII), IncFIB(AP001918)
Cluster 22	1D-139 1D-845^a^	12	461	96.8	ST-69	**Blood** **Urine**	**F5** **F3**	***parC* S80I, *tet*(A) (only 1D-845)**	**IncFIA, IncFII(pCoo) (only 1D-139), IncI1-I(Alpha) (only 1D-845), IncFIC(FII) (only 1D-845), Col(MG828) (only 1D-845), IncFIB(AP001918) (only 1D-845)**
Cluster 23	1D-551 1D-552^a^	14	0	99.1	ST-453	Blood	F1	None	None

Text in bold indicates different sample sources, DCM laboratory, ARGs or plasmids in the same cluster. Plasmid replicons separated with ‘/’ were identified in the same contig.

^a^Indicates which genome was used as reference for SNP-based cluster analysis with CSI Phylogeny.

SNP-based phylogenetic analysis of the 23 clusters identified through cgMLST revealed that, within most clusters (*n* = 19), the distances between isolates were below two SNPs, including in the three clusters composed of more than two isolates (Table S5). In two additional clusters, the SNP distances were, respectively, 12 and 13 SNPs (clusters 14 and 16). In the remaining two clusters, the distances were much larger, with 111 and 461 SNPs (clusters 12 and 22). In all clusters, regardless of SNP distance, at least 95.3% of the reference genome was used in the alignment with the query genomes, with an average of 99.2% of alignment throughout all clusters, and with the vast majority (*n* = 19) showing percentages of alignment over 99% (Table 3).

## Discussion

In this study we analysed the genomes of all *E. coli* isolates (*n* = 699) processed in all DCM laboratories during 1 day. We observed that the percentages of isolates harbouring ARGs or PMs conferring resistance to specific antimicrobial agents were similar to the percentages of phenotypic resistance described in the Danish AMR surveillance reports, in the same and the following year, in urine and invasive *E. coli* isolates in the country.^37,38^ From the national surveillance data in 2018 and 2019, approximately 40% of Danish isolates were phenotypically resistant to ampicillin, in concordance with our findings of 40.1% of *E. coli* isolates harbouring ARGs or PMs conferring resistance to β-lactam antimicrobials. The percentages of isolates presenting genetic determinants of resistance to sulphonamides (31.9%), trimethoprim (28.3%) and gentamicin (4.1%) were also in agreement with the percentages of phenotypic resistance to those antimicrobials described in the same surveillance reports (approximately 30%, 24% and 4.5%, respectively). The agreement between AMR prevalence in national surveillance data and those calculated in this study through genotypic analysis (Table 2) suggests that *in silico* AMR predictions are useful to estimate national prevalence of phenotypic resistance to, at least, those antimicrobials. The exception was that the genetic determinants of ciprofloxacin resistance detected in this study were much more abundant (22.2%) than the effective national prevalence of resistant isolates (∼10%). This variation can be due to the fact that single PMs in gyrase and topoisomerase genes increase the MIC of ciprofloxacin in *E. coli*, but often not enough to place them in the clinically resistant concentration range.^39,40^ Similarly, certain ARGs are associated with increases in MIC that might not be high enough to lead to classification as clinically resistant, such as *qnr* genes and *aac(6′)-Ib-cr.*^41–43^ These gradual increases in MIC derived from the accumulation of PMs or caused by specific ARGs must be considered when designing bioinformatics tools or pipelines to automatically predict phenotypic resistance from genomic data. This observation highlights that the detection of ARGs or PMs is not always perfectly associated with the expected phenotypes, as also shown in multiple other studies.^44–48^ According to conclusions from our previous phenotype–genotype concordance study, where we used the same sequencing and bioinformatics strategies and the same sequence quality thresholds, we predict that 72% of all Danish *E. coli* isolates harbouring genetic AMR determinants are phenotypically resistant to the respective antimicrobials.^49^ Thus, improvement in reference databases is essential in order to allow for prediction of individual resistance profiles of *E. coli* isolates. This includes, for example, ensuring that the databases remain updated according to the most current knowledge of genetic mechanisms of AMR, ensuring that the association of those determinants with specific AMR phenotypes is done at the individual antimicrobial level and not only at class level, and including warnings when there are cumulative effects between genetic determinants of AMR and when the genetic determinants by themselves might not provoke a sufficient increase in MIC to place the isolates in the clinical resistance range.

The most prevalent MLSTs detected in this study (ST-69, ST-73, ST-95 and ST-131) correspond to those also frequently detected worldwide.^50–52^ There was no overall clustering of isolates according to geographical origin or sample source, showing that the different *E. coli* STs are distributed throughout the country and not restricted to a particular region (Figure 2). These results are in agreement with conclusions from other studies conducted in Denmark, reporting high prevalence of the same STs, and additionally ST-38, in uropathogenic *E. coli* with varied AMR profiles and in *E. coli* from bloodstream infections resistant to third-generation cephalosporins.^53,54^ Danish surveillance data from 2018 to 2020 show that ST-131 was the most common MLST ST identified among plasmid-mediated AmpC- or ESBL-producing *E. coli* recovered from bloodstream infections (approximately 50% of those isolates). These surveillance data also reveal possible or verified outbreaks of carbapenemase-producing *E. coli* attributed to ST-38, ST-73, ST-167, ST-410 and others.^37,38,55^ These observations support the importance of accurate MLST determination and adequate cluster analysis in the national clinical context.

cgMLSTFinder was able to assign alleles to a high (80%–90%) or very high (>90%) percentage of the 2513 loci in the scheme for most isolates (*n* = 696; 99.6%). We defined an exploratory dissimilarity of 15 alleles as the threshold to classify isolates as being genetically related, which yielded 23 clusters. All isolates in each cluster shared the same MLST, supporting the applicability of cgMLST for typing of *E. coli* recovered from clinical settings. SNP-based analysis of isolates in each cluster revealed very small differences between genomes, with the majority of clusters presenting <2 SNPs and two additional clusters presenting 12 and 13 SNPs, respectively. These distances are consistent with other studies, besides those previously described, revealing distances of 5, 7 and 13 SNPs between epidemiologically related *E. coli* clones within nosocomial or community-associated outbreaks, including in Denmark.^54,56,57^ However, two cgMLST-based clusters presented much larger differences, with 111 and 456 SNPs. We propose that the isolates in these clusters probably do not exhibit a clonal relationship between themselves, and that the high number of SNPs illustrates the higher discriminatory power of SNP-based analysis when compared with gene-by-gene methods, for closely related isolates. Probably, the positions with nucleotide variations fall outside the loci included in the cgMLST scheme, thus not being adequately captured by that approach. This emphasizes the importance of continuous benchmarking of sequencing and bioinformatics approaches using sequencing datasets with well-defined expected results, in the laboratories where they are being employed, to confirm if those approaches are able to properly identify relevant clusters.

Distribution of ARGs within clusters was also consistent, except for one of the clusters where SNP-based analysis revealed a high SNP difference, further supporting the choice of very conservative thresholds for cgMLST analysis and the importance of confirming genetic relatedness through more discriminatory methods. Plasmid distribution was more heterogeneous, with four cases where only one member of the cluster harboured certain plasmid replicons. This can be a true reflection of variability in mobile genetic elements of genetically related isolates, for example due to plasmid loss or variations in copy number.^58^ However, the possibility that these are artefacts derived from the DNA extraction laboratory protocol or from the bioinformatics processes used in this study cannot be excluded, since these are optimized for recovery and analysis of chromosomal DNA.^59,60^

Due to data protection and patient privacy laws, we did not have access to any patient metadata. Thus, it is not possible to draw any conclusions about the epidemiological significance of the detected clusters, especially those with isolates from the same DCM laboratory and the same sample source. The isolates might, for example, originate from different samples collected from the same patient, or might originate from the same sample that has been cultured in several agar plates, therefore not corresponding to transmission between patients or potential outbreaks. The lack of this information makes it difficult to predict the adequacy of the selected clustering thresholds, although the overall concordance of cgMLST- and SNP-based analysis supports their appropriateness. This limitation highlights the value of associating adequate metadata and epidemiological data with WGS results, to allow for the creation of actionable information to guide clinical treatment, infection prevention and control measures, and surveillance decisions.

In conclusion, we have shown that WGS provides high-quality data, which can be used to accurately perform multiple types of analysis, in particular species identification, prediction of AMR and plasmid content, bacterial typing and clustering analysis. Detection of ARGs and PMs had good correlation with national prevalence of phenotypic resistance, suggesting that WGS-based analysis can be used for monitoring national resistance trends and control emergence and dissemination of known AMR mechanisms. However, some reservations remain regarding the immediate applicability of WGS to predict individual resistance profiles. Furthermore, bacterial subtyping through WGS leads to STs in concordance with those detected through national surveillance systems and in other countries. Finally, clustering results are accurate and may have the potential to lead to faster resolution of outbreaks, with decreases in burden of disease and improvement of public health surveillance systems. Expanding reference gene databases, benchmarking specific bioinformatics approaches and defining global and harmonized interpretative thresholds are steps that will strengthen the outcomes of these analyses and allow for their full implementation in clinical microbiology laboratories.

## Supplementary Material

dkaf028_Supplementary_Data

## Supplementary data

Figure S1 and Tables S1–S6 are available as Supplementary data at *JAC* Online.
